# Mitochondrial Inhibition by Sodium Azide Induces Assembly of eIF2α Phosphorylation-Independent Stress Granules in Mammalian Cells

**DOI:** 10.3390/ijms23105600

**Published:** 2022-05-17

**Authors:** Nina Eiermann, Georg Stoecklin, Bogdan Jovanovic

**Affiliations:** 1 Division of Biochemistry, Mannheim Institute for Innate Immunoscience (MI3), Mannheim Cancer Center (MCC), Medical Faculty Mannheim, Heidelberg University, 68167 Mannheim, Germany; nina.eiermann@medma.uni-heidelberg.de (N.E.); georg.stoecklin@medma.uni-heidelberg.de (G.S.); 2 Center for Molecular Biology of Heidelberg University (ZMBH), DKFZ-ZMBH Alliance, 69120 Heidelberg, Germany; 3 Center for Human Molecular Genetics, Faculty of Biology, University of Belgrade, 11000 Belgrade, Serbia

**Keywords:** stress granules, integrated stress response, translation, mitochondrial stress, sodium azide

## Abstract

Mitochondrial stress is involved in many pathological conditions and triggers the integrated stress response (ISR). The ISR is initiated by phosphorylation of the eukaryotic translation initiation factor (eIF) 2α and results in global inhibition of protein synthesis, while the production of specific proteins important for the stress response and recovery is favored. The stalled translation preinitiation complexes phase-separate together with local RNA binding proteins into cytoplasmic stress granules (SG), which are important for regulation of cell signaling and survival under stress conditions. Here we found that mitochondrial inhibition by sodium azide (NaN_3_) in mammalian cells leads to translational inhibition and formation of SGs, as previously shown in yeast. Although mammalian NaN_3_-induced SGs are very small, they still contain the canonical SG proteins Caprin 1, eIF4A, eIF4E, eIF4G and eIF3B. Similar to FCCP and oligomycine, other mitochodrial stressors that cause SG formation, NaN_3_-induced SGs are formed by an eIF2α phosphorylation-independent mechanisms. Finally, we discovered that as shown for arsenite (ASN), but unlike FCCP or heatshock stress, Thioredoxin 1 (Trx1) is required for formation of NaN_3_-induced SGs.

## 1. Introduction

Unicellular organisms and cells in multicellular organisms encounter many external and internal agents, some of which may induce stress. Stress represents a condition that negatively affects cellular growth and may lead to pathological conditions or cell death. All types of stress lead to the damaging of lipids, nucleic acids and proteins in cells [1]. Therefore, during evolution, it was essential for cells to develop different strategies to sense stress, transmit information and elicit an appropriate adaptive response. Adaptation to sudden changes in the cellular environment is mostly achieved through changes in gene expression, which are necessary to survive exposure to different environmental stresses and re-establish cellular homeostasis [2,3]. The regulation of translation has an advantage over transcriptional control, since drawing on pre-existing mRNA allows for faster changes in cellular protein concentrations [2]. The integrated stress response (ISR) is an evolutionarily conserved, protective molecular signaling mechanism that enables cells to cope with stress and maintain homeostasis [4]. The key players in the ISR are four kinases (GCN2, HRI, PERK, PKR), each for sensing a different type of stress, with the common substrate eukaryotic translation initiation factor 2 alpha (eIF2α) [4,5]. The phosphorylation of eIF2α on serine (S)51 converts the factor from a substrate to a competitive inhibitor of its guanidine exchange factor eIF2B [2,6]. As a result, the impaired nucleotide exchange blocks protein synthesis by preventing the recycling of inactive eIF2α-GDP to functional eIF2α-GTP, which is required for the delivery of the initiator Met-tRNAi to the 40S ribosomal subunit during translation initiation [2,6]. A consequence of stalled translation initiation is the formation of cytoplasmic stress granules (SGs) through a phase-separation process [7,8,9,10]. These granules are multimolecular aggregates of stalled translation pre-initiation complexes, small ribosomal subunits and locally present RNA-binding proteins (RBPs) [10,11]. The biological function of SGs is not yet fully understood, but they are proposed to be sites of mRNA storage and triage that allow a fast reinitiation of translation when stress is resolved [12]. Besides RBPs, SGs also contain other proteins that do not bind RNA directly, but which are recruited to SGs via protein–protein interactions [13,14,15]. The proteome of SGs shows variations according to stress type and level of stress [13,14,16], and the functions of SGs might differ accordingly. Since both pro-apoptotic and anti-apoptotic factors have been found within SGs, they may be able to determine the cellular fate under stress conditions [17,18,19]. In addition, SGs sequester other signaling proteins and enzymes and may thus serve as general signaling platforms that aid in the coordination of stress and immune signaling pathways [7]. As SGs are stress-specific, their characterization might help in obtaining a better understanding of the cellular response to hazardous stress or pathological states. 

The mechanisms and consequences of SG formation upon mitochondrial inhibition are rather complex, and conflicting findings have been reported in the literature. As mitochondria are essential organelles, and mitochondrial dysfunction is a driver of pathogenesis, it is important to untangle the underlying processes of the SG response to mitochondrial stress. Oligomycine and mitochondrial uncouplers such as Carbonyl cyanide-p-trifluoromethoxyphenylhydrazone (FCCP) and Carbonyl cyanide m-chlorophenyl hydrazone (CCCP) were shown to activate HRI kinase [20,21]. However, Kedersha et al. observed that FCCP and oligomycine induced SGs independently of HRI activation and eIF2α phosphorylation [22]. Later, we confirmed these findings, showing that HRI is not important for SGs formed upon FCCP treatment [23]. Mitochondrial complex I inhibition was not investigated in terms of the SG formation, but HRI kinase is thought to mediate a stress response upon exposure to rotenone, which inhibits the mitochondrial complex I [21]. Malonate, a competitive inhibitor of the mitochondrial electron transport chain complex II, was found to induce eIF2α-independent, non-canonical SGs through a mechanism involving the translation inhibitor 4E binding protein (BP) 1 [24]. Antimycin A, an inhibitor of complex III and inducer of oxidative stress, was found to lead to an increase in eIF2α phosphorylation and a decrease in mTOR activity [25]. However, complex III inhibition has not been explored in respect to SG formation.

Carbon monoxide (CO), which inhibits mitochondrial complex IV, triggers HRI kinase activation [26]. Another study showed that CO also activated PERK, which was needed for SG formation upon exposure to CO [27]. Sodium azide (NaN_3_) is another inhibitor of mitochondrial complex IV [28,29]. Its effect has been mainly investigated in yeast, with a SG response differing considerably from mammals [14,30,31]. In these studies, NaN_3_ strongly inhibited translation and induced SGs in an unknown, but eIF2α phosphorylation-independent, manner [14,31]. To our knowledge, there is only one study to date about the NaN_3_-induced stress response in mammalian cells, showing the formation of NaN_3_-induced SGs in rat renal tubular cells [32]. In accordance with the reports from yeast, the authors of this study found eIF2α to be phosphorylated. However, it remains unclear whether the observed SG induction in response to NaN_3_ was mediated via eIF2α phosphorylation. 

In the study presented here, we further characterize SG formation upon mitochondrial inhibition that is caused by NaN_3_ treatment in different mammalian cell types, thereby expanding our knowledge concerning: SG composition; SG kinetics in relation to translational inhibition and signaling; and the implications of eIF2α phosphorylation and other factors for the formation of SGs.

## 2. Results

### 2.1. Mitochondrial Inhibition by NaN_3_ Is a Potent Trigger for SG Formation in Mammalian Cells

**Figure 1:**
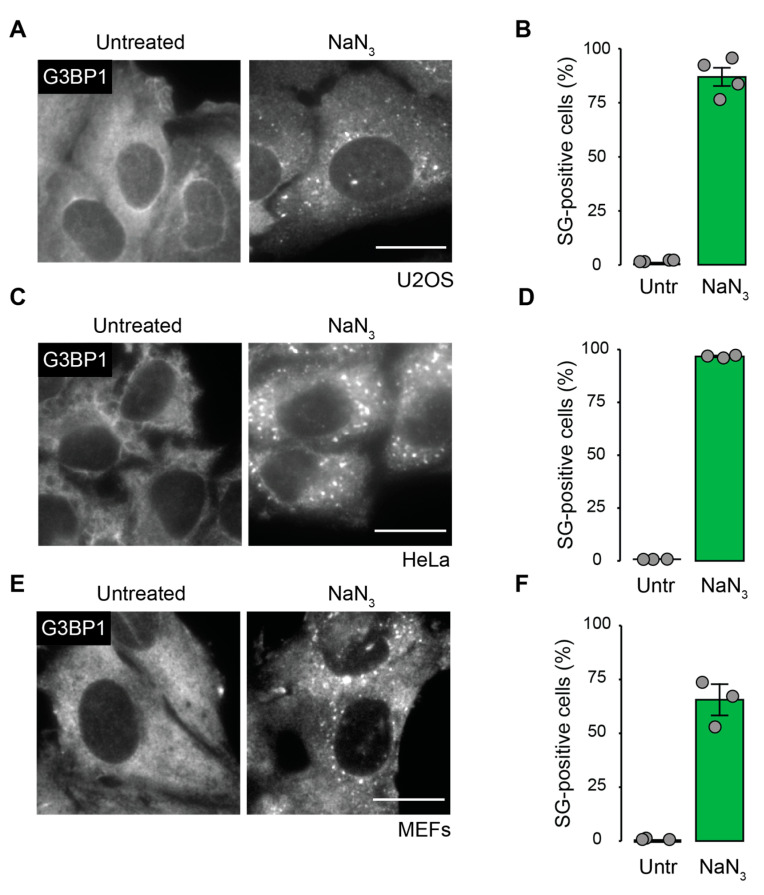
The inhibition of mitochondrial complex IV by NaN_3_ induced SG formation in mammalian cells. Cells were left untreated or treated with NaN_3_ for 2 h, then fixed and stained with anti-G3BP1 antibody for the detection of SGs. (**A**) SG formation in U2OS cells; scale bar = 20 µm. (**B**) Quantification of A. (**C**) SG formation in HeLa cells; scale bar = 20 µm. (**D**) Quantification of C. (**E**) SG formation in MEFs; scale bar = 20 µm. (**F**) Quantification of E. All graphs show mean value ± SD (*n* ≥ 3 independent experiments; approximately 100 cells were analyzed per experiment and condition).

The cellular SG response to mitochondrial stress caused by the inhibition of mitochondrial complex IV has not been investigated in mammalian systems in great detail. To better understand the effect of SG assembly upon mitochondrial stress, we treated U2OS cells with NaN_3_ and monitored the SG formation by an immunofluorescence (IF) analysis of G3BP Stress Granule Assembly Factor 1 (G3BP1), a well-known SG marker [33,34]. The treatment conditions were the same as in yeast studies (a concentration of 0.5% *v/v* corresponding to 76 mM NaN_3_, using a low-glucose medium during treatment) [14] and always compared to control conditions where cells were kept in a low-glucose medium for the same amount of time but without the addition of NaN_3_. We performed MitoTracker staining (CM-H_2_TMRos) and confirmed that our treatment conditions led to a severe inhibition of mitochondrial activity (Figure S1). As a consequence, NaN_3_ effectively induced SG assembly in U2OS cells, HeLa cells and MEFs (Figure 1). About 86% of U2OS and 66% of MEFs formed SGs after 2 h of NaN_3_ treatment. The strongest response was seen in HeLa cells, where almost all cells formed SGs. Surprisingly, the observed SGs displayed an unusually small size in all tested cell lines. In the case of HeLa cells, NaN_3_ treatment led to the formation of SGs with an average diameter of 0.19 µm (median value 0.15 µm), which is 15 times less than the average diameter of arsenite (ASN)-induced SGs (2.85 µm, median value 2.2 µm, *p* < 0.000001) (Figure S2A,B). Treatment in a high-glucose medium reduced the number of SGs by approximately fourfold, implying that ATP depletion contributed to SG formation (Figure S2C). 

**Figure 2:**
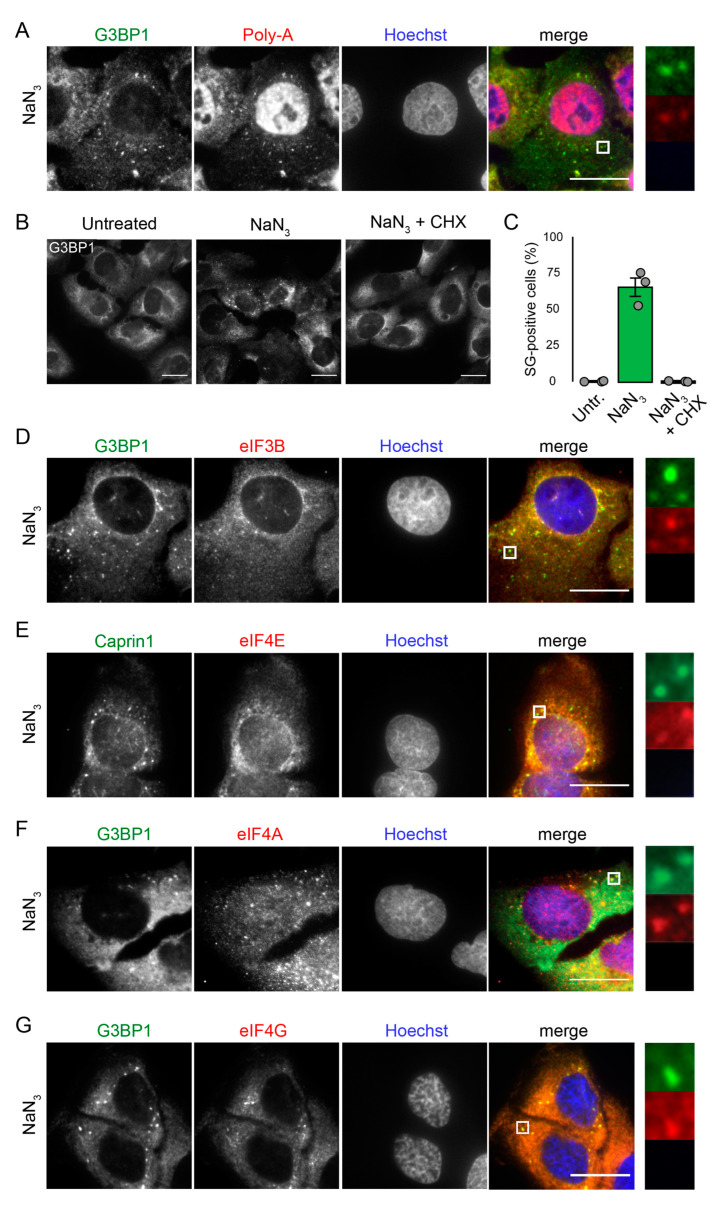
Detailed characterization of NaN_3_-induced SGs. (**A**) Fluorescence in situ hybridization with an oligo-(dT) probe was used to visualize mRNA in U2OS cells that were treated with NaN_3_ (76 mM) for 1 h. Cells were stained with anti-G3BP1 antibody. Subcellular localization of G3BP1 was used for the detection of SGs; Scale bar = 20 µm. (**B**) Cells were left untreated, treated with NaN_3_ (76 mM), or CHX and NaN_3_ in combination. (**C**) A quantification of SG-positive cells in B is presented. The graph shows the mean value ± SD (*n* = 3 independent experiments; approximately 100 cells were analyzed per experiment and condition). (**D**) Co-immunostaining of eIF3B and G3BP1 in U2OS cells treated with NaN_3_; Scale bar = 20 µm. (**E**) Co-immunostaining of Caprin1 and eIF4E in U2OS cells treated with NaN_3_; Scale bar = 20 µm. (**F**) Co-immunostaining of eIF4A and G3BP1 in U2OS cells treated with NaN_3_; Scale bar = 20 µm. (**G**) Co-immunostaining of eIF4G and G3BP1 in U2OS cells treated with NaN_3_; Scale bar = 20 µm.

Next, we explored the composition of NaN_3_-induced SGs. In addition to the presence of well-known SG markers such as G3BP1, fluorescence in situ hybridization with an oligo-(dT) probe confirmed the presence of mRNA within the observed aggregates (Figure 2A). Furthermore, cycloheximide (CHX) treatment, which interferes with polysome disassembly, prevented the formation of the granular structures (Figure 2B,C), a feature that is typical for SGs. The immunostaining of NaN_3_-treated cells for eIF3B, a marker for canonical SGs [16,35], confirmed the co-localization of eIF3B with G3BP1 (Figure 2D), indicating that NaN_3_ induced canonical SGs by composition. By staining against eIF3B alone, we excluded a possible “bleed-through” artifact (Figure S3A). Similar to ASN-induced SGs [36,37] (Figure S3B–E), NaN_3_-induced SGs contain Caprin1 and many additional translation initiation factors such as eIF4E, eIF4A and eIF4G (Figure 2E–G).

### 2.2. Kinetics of NaN_3_-Induced SG Formation Coincide with Translation Inhibition and Stress Signaling

**Figure 3:**
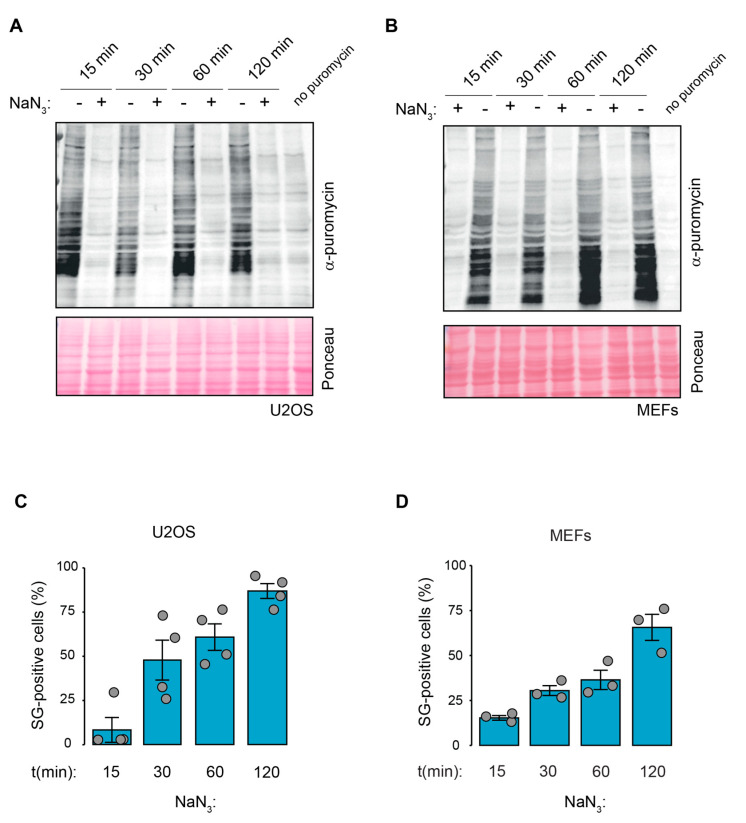
Kinetics of the NaN_3_-induced SG response and translation inhibition. (**A**,**B**) Protein synthesis in U2OS cells (**A**) and MEFs (**B**) exposed to NaN_3_ (76 mM) was measured at different time points upon NaN_3_ addition using a WB-based puromycin incorporation assay. Ponceau staining served as the loading control. As a control, a sample without puromycin labeling was also loaded. (**C**,**D**) U2OS cells (**C**) and MEFs (**D**) were treated with NaN_3_ (76 mM) at different time points as indicated. Cells were fixed, stained against G3BP1 and analyzed using IF. The number of SG positive cells was quantified using visual inspection. The graphs show the mean value ± SD (*n* ≥ 3 independent experiments; approximately 100 cells were analyzed per experiment and condition).

**Figure 4:**
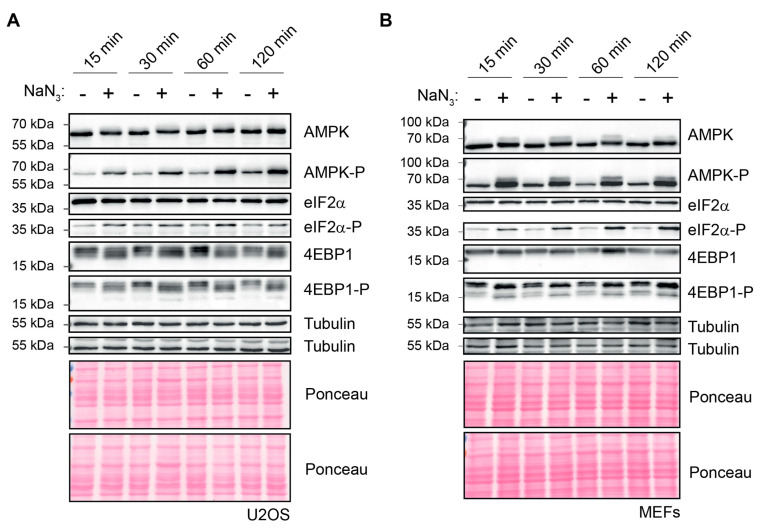
Altered cellular signaling in response to NaN_3_ exposure. (**A**) U2OS cells were left untreated or subjected to NaN_3_ stress (76 mM) of different durations. Cells were lysed, and the lysates were immunoblotted against the indicated proteins to investigate the activation of different signaling pathways. Tubulin and Ponceau stainings served as loading controls. (**B**) MEFs were cultivated under normal conditions or subjected to NaN_3_ stress (76 mM) of different durations and prepared for WB the same as in (**A**).

SG formation is typically a downstream consequence of translation inhibition, frequently mediated via the phosphorylation of eIF2α [7]. To monitor the NaN_3_-induced stress-response kinetics, we measured SG assembly in MEF and U2OS cells over time in parallel to changes in translation and the activation of stress signaling pathways. Puromycin incorporation assays showed that translation was strongly suppressed already after 15 min of NaN_3_ treatment in both human and murine cell lines (Figure 3A,B). In contrast to the early and immediate inhibition of translation, the formation of SGs increased progressively over time in both U2OS cells and MEFs (Figure 3C,D). Whereas only 8% of the U2OS cells and 15% of the MEFs contained SGs after 15 min of NaN_3_ treatment, the proportion rose to 86% (U2OS) and 65% (MEFs) after 2 h. Along with the formation of SGs and translation inhibition, a Western blot (WB) analysis of extracts from NaN_3_-treated cells revealed multiple changes in the stress-response signaling pathways (Figure 4A,B). First, we observed increased eIF2α phosphorylation upon NaN_3_ treatment. In U2OS, eIF2α phosphorylation was detected after 15 min, and the phosphorylation levels remained the same during the exposure period of 2 h. Similarly, in MEFs, eIF2α phosphorylation was also detected after 15 min, but the levels gradually increased during the course of 2 h. Second, AMPK, which responds to reduced cellular energy levels, was found to be phosphorylated at T172, reflecting the activation of the kinase. The phosphorylation of AMPK had occurred already after 15 min exposure to NaN_3_, and it progressively increased in U2OS cells. Third, 4EBP1 was found to be dephosphorylated, which occurred through the inhibition of mTOR signaling and converted 4EBP1 into an inhibitor of cap-dependent translation [38]. 

### 2.3. NaN_3_-Induced SGs Are Assembled Independently of eIF2α Phosphorylation

**Figure 5:**
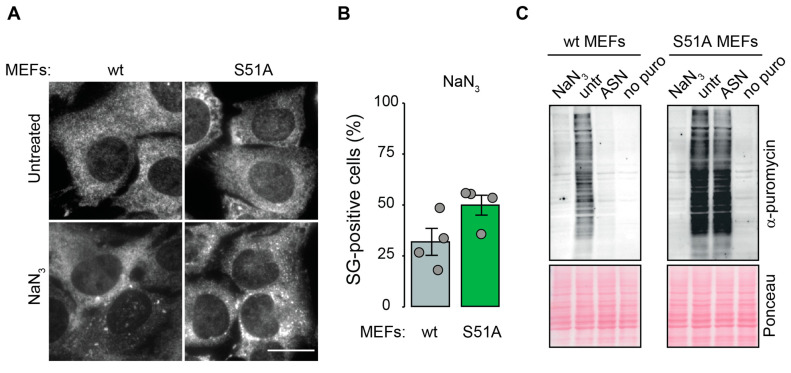
SGs formed upon NaN_3_ exposure do not require eIF2α phosphorylation. (**A**) MEFs were left untreated or treated with NaN_3_ for 1 h before fixation. Fixed cells were stained with anti-G3BP1 antibody for IF microscopy, and SG-containing cells were quantified by visual inspection; Scale bar = 20 µm. (**B**) Quantification of A. The graphs show the mean value ± SD (*n* = 4 independent experiments; approximately 100 cells were analyzed per experiment and condition). (**C**) Measurement of protein synthesis using a puromycin incorporation assay in MEFs treated with NaN_3_ for 1 h, treated with ASN (500 µM) for 1 h, or left untreated. As a control, a sample without puromycin labeling was also loaded.

The phosphorylation of eIF2α plays a central role in the induction of SGs under acute stress conditions [9,39]. Since we observed elevated eIF2α phosphorylation following exposure to NaN_3_, we wanted to investigate the importance of eIF2α phosphorylation for NaN_3_-induced SGs. To this end, we utilized eIF2α-S51A MEFs carrying a phospho-deficient mutation in both alleles of the endogenous eIF2α gene [40], which are unable to suppress translation and to trigger SG assembly under different conditions, such as ASN-induced oxidative or ER-stress [41,42]. As expected, eIF2α phosphorylation was not detected in S51A MEFs (Figure S4A), but, surprisingly, both wildtype (wt) and S51A MEFs formed SGs upon exposure to NaN_3_ (Figure 5A,B). Likewise, puromycin incorporation assays showed that NaN_3_, unlike ASN, inhibited translation to a similar level in both WT and S51A MEFs (Figure 5C). To validate the observation that eIF2α phosphorylation is not necessary for the observed SG response, we used knockout (ko) MEF cell lines, each lacking one of the four eIF2α kinases. In agreement with the results from the S51A MEFs, the PKR^−/−^, PERK^−/−^, GCN2^−/−^ and HRI^−/−^ MEFs were all able to form SGs in response to NaN_3_ treatment (Figure S4B). 

### 2.4. Trx1 Depletion Prevents Formation of NaN_3_-Induced SGs

**Figure 6:**
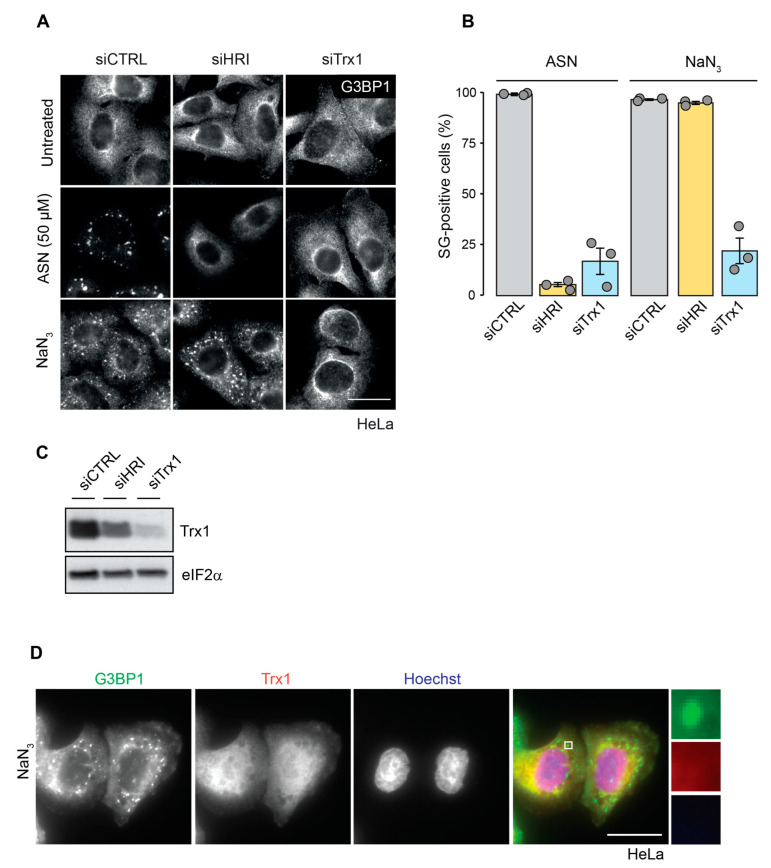
Trx1 is required for the formation of SGs under NaN_3_-induced stress. (**A**) HeLa cells were transfected with the non-targeting control siRNA, or an siRNA targeting HRI, or Trx1 for 72 h, treated with 50 µM ASN for 1 h, or 76 mM NaN_3_ for 2 h, and prepared for IF microscopy; Scale bar = 20 µm. (**B**) Quantification of A. The graph shows the mean value ± SD (*n* = 3 independent experiments; approximately 100 cells were analyzed per experiment and condition). (**C**) Knockdown of Trx1 was assessed with a WB analysis; eIF2α served as loading control. (**D**) HeLa cells were treated with 50 µM ASN for 1 h or 76 mM NaN_3_ for 2h. Fixed cells were stained with anti-G3BP1 and anti-Trx1 antibodies for IF microscopy. Hoechst staining was used for the detection of nuclei; Scale bar = 20 µm.

In addition to translation inhibition acting as a main trigger for SG formation, a growing number of RBPs and enzymes have been identified to regulate the assembly, disassembly and dynamics of SGs. Recently, we identified Trx1, a major antagonist of cellular protein oxidation, as a novel component of ASN-induced SGs that is involved in SG assembly [23]. Trx1 depletion prevented the formation of SGs upon ASN-induced oxidative stress, but not upon heat shock or mitochondrial inhibition by FCCP [23]. To test whether Trx1 might play a role in the formation of NaN_3_-induced SGs, we performed a knockdown of Trx1 and monitored the SG formation upon exposure to NaN_3_. HeLa cells were used because siRNA transfection is very efficient when used in this system. For comparison, cells were also treated with ASN and subjected to HRI knockdown, which is known to prevent SG assembly upon ASN treatment. Trx1 knockdown efficiently reduced Trx1 expression levels and, as expected, prevented the formation of ASN-induced SGs (Figure 6A–C). To our surprise, the knockdown of Trx1 also prevented SG formation upon NaN_3_ exposure. While 96% of cells transfected with control non-targeting siRNA formed SGs, only 22% of Trx1-depleted cells formed SGs under NaN_3_ stress conditions (*p* < 0.0001).

Finally, we investigated whether Trx1 localizes to NaN_3_-induced SGs by performing an IF analysis. However, we did not observe any co-localization of Trx1 and G3BP1 in SGs (Figure 6D), indicating that Trx1 might act on the proteins that are important for SG formation outside of SGs, and thereby affect the assembly of SGs under NaN_3_-induced mitochondrial stress. 

## 3. Discussion

Depending on the cause of mitochondrial stress, the pathways that are activated and therefore, the implications for the cell, can differ. Mitochondrial stress, caused by NaN_3_ treatment, which inhibits complex IV, induced SG formation at early time points in both human and murine cell lines (Figure 1). 

The observed stress response is similar to that in yeast, where NaN_3_ strongly induces SG assembly [14]. However, while the SGs in yeast are of a similar size to those observed in other SG-inducers such as ASN [14], NaN_3_-induced SGs in mammalian cells are much smaller than typical SGs. In HeLa cells, the average diameter of SGs formed upon NaN_3_ treatment was 15 times smaller compared to SGs induced by ASN exposure (Figure S2). Typical SGs are characterized by their growth during the stress response, which involves the fusion of small granules with each other or with P-bodies, which are condensates containing RNA-processing enzymes [8]. This fusion process happens in a microtubule-dependent manner and requires ATP [43]. Since NaN_3_ inhibits mitochondrial complex IV and F0F1 ATPase, leading to ATP depletion [29,44,45,46], this could explain why NaN_3_-induced SGs are much smaller in size. SGs observed upon treatment with FCCP or oligomycine [22], however, are not that small, indicating that other factors also influence the size of SGs.

The immunofluorescence microscopy of the U2OS cells revealed the composition of NaN_3_-induced SGs. They contain mRNA in addition to two well-known SG markers, G3BP1 and Caprin1 [37], indicating that the granules are *bona fide* SGs (Figure 2). Consistent with the presence of mRNA, CHX treatment, which inhibits the disassembly of polysomes and thereby the accumulation of stalled translation initiation complexes, prevented NaN_3_-induced SG assembly (Figure 2). The SGs were also positive for eIF3B, another marker for canonical SGs in mammals (Figure 2), which is consistent with eIF3B stainings of NaN_3_-treated rat renal tubular cells [32]. Like in yeast [14], translation initiation factors eIF4A, eIF4G and eIF4E were recruited to mammalian NaN_3_-induced SGs (Figure 2), indicating the sequestration of stalled translation pre-initiation factors.

In line with the detection of SGs within mammalian cells upon NaN_3_ treatment, we observed a massive inhibition of global protein synthesis preceding SG formation. Whereas translation inhibition occurred as early as 15 min after NaN_3_ exposure, SG formation increased gradually over time as a consequence of translation inhibition (Figure 3). In parallel to the early suppression of translation, multiple signaling cascades were affected upon NaN_3_ treatment, including the activation of AMPK kinase, mTOR inhibition and eIF2α phosphorylation (Figure 4). The phosphorylation of eIF2α is a key event in the ISR and is responsible for the formation of SGs under most types of stress [5]. In the case of NaN_3_ exposure, the S51A MEFs expressing non-phosphorylatable eIF2α showed that eIF2α phosphorylation was not required for SG formation (Figure 5 and Figure S4). This is similar to NaN_3_-induced SGs in yeast, which also form independently of eIF2α phosphorylation [14]. It is further in line with other mitochondrial stressors, such as the uncoupling agent FCCP or oligomycine, which also induce phospho-eIF2α-independent SGs in mammalian cells [22]. Our observation was additionally strengthened by the fact that the ko MEFs lacking the individual eIF2α kinases were still able to form SGs upon NaN_3_ exposure (Figure S4). Noticeably, these data show that PERK is not important for NaN3-induced SGs, although PERK has been reported to be activated in response to NaN_3_ treatment [32]. Hence, alternative and/or parallel signaling pathways seem to control translation inhibition and SG assembly upon NaN_3_ treatment in an eIF2α-phosphorylation independent manner. As eIF2α phosphorylation is not required, translation suppression and SG formation are also probably independent from the ISR in general. Interestingly, the phosphorylation of AMPK and inhibition of mTOR signaling were shown to contribute to translation arrest and SG assembly under conditions of cold shock [47]. Since mitochondrial dysfunction by NaN_3_ treatment leads to a similar drop in ATP levels and AMPK phosphorylation, it would be interesting to further explore the role of AMPK signaling in translation inhibition and SG assembly. 

Finally, we discovered that the depletion of Trx1 prevented the formation of SGs under mitochondrial stress caused by NaN_3_ exposure (Figure 6). This is similar for ASN-induced SGs, but different from FCCP-induced SGs, where Trx1 depletion had no effect on SG formation [23]. Unlike in ASN-induced SGs, Trx1 was absent from SGs formed upon NaN_3_ treatment [23]. Therefore, Trx1 activity is most likely needed for the proper function of another SG assembly factor upon complex IV inhibition by NaN_3_. Out of all thioredoxin paralogs, only Thioredoxin 2 (Trx2) is present in mitochondria, where it provides protection against high ROS levels [48]. However, there is evidence highlighting the importance of Trx1 for mitochondrial homeostasis and health [49,50]. As we identified Trx1 to be essential for SG assembly upon NaN_3_ treatment, and since SGs are thought to regulate cell survival [9,23], our results might provide a clue to the previous results showing that Trx1 is important for survival upon mitochondrial damage [50]. Given the connection between mitochondrial homeostasis and redox signaling, and considering that mitochondrial stress is associated with many pathological conditions [51], it might be interesting and useful to further explore the connections between mitochondrial stress, SG formation and the Trx1 system.

### Conlusion

Within different mammalian cell types, we demonstrated that mitochondrial stress caused by NaN_3_ triggered translation inhibition and the formation of tiny SGs with a similar composition as in yeast. NaN_3_-induced SGs in mammals are induced in an eIF2α phosphorylation-independent manner, which is similar to yeast and to SGs formed by other types of mitochondrial inhibitors such as FCCP and oligomycine. Our identification of Trx1 as a factor required in mammalian cells for SG formation upon NaN_3_ treatment highlights a connection between redox signaling, mitochondrial stress and SG formation. 

## 4. Materials and Methods

### 4.1. Cell Culture

HeLa, U2OS and MEF cells were cultured in Dulbecco’s modified Eagle’s medium (DMEM, Gibco, Waltham, MA, USA) supplemented with 10% fetal bovine serum (Sigma, St. Louis, MO, USA), 2 mM L-glutamine, 100 U/mL penicillin and 0.1 mg/mL streptomycin (all PAN Biotech). Cells were grown at 37 °C and 5% CO_2_. Where indicated, cells were treated with ASN (NaAsO_2_, Sigma) or NaN_3_ (AppliChem, Omaha, NE, USA). During treatment and 2h before treatment, cells were cultivated in DMEM with 1g/l D-Glucose (low-glucose) (Gibco). In parallel, control cells were kept in a low-glucose medium without NaN_3_. 

### 4.2. Transfection of Cells

Knockdown experiments were performed using Lipofectamine RNAiMAX transfection reagent (Thermo Fisher) according to the manufacturer’s protocols. A full 72 h after transfection, cells were processed for analysis. siRNA s119 (5′-GCAGCGAUCUGAUGAAUUG-3′) was used for kd of HRI; siRNA s117 (5′-AUGACUGUCAGGAUGUUGC-3′) was used for kd of Trx1; a non-specific siRNA C2 (5′-GGUCCGGCUCCCCCAAAUG-3′) served as a control. 

### 4.3. Immunofluorescence Analysis

Cells were seeded onto glass coverslips one day before drug treatment. Cells were fixed and permeabilized with ice-cold methanol for 3 min, washed with PBS and blocked with 3% BSA in PBS for 1 h at room temperature. Alternatively, cells were fixed with 4% paraformaldehyde in PBS for 15 min followed by 10 min post-permeabilization with 0.5% Triton-X in PBS. 

Coverslips were then incubated with the appropriate primary antibodies diluted in PBS containing 0.1% NaN_3_ (PBS-A) for 1 h rocking at room temperature or overnight at 4 °C. Cells were washed with PBS three times for 5 min before Cy2- or Cy3-coupled secondary antibodies (Jackson ImmunoResearch, West Grove, PA, USA) that were diluted 1:1000 in PBS and Hoechst dye (1:10,000, Sigma) were added for 1 h at room temperature. After washing again three times for 5 min with PBS, coverslips were mounted onto glass slides using FluoroG mounting medium (Thermo Fisher, Waltham, MA, USA). Microscopy was performed on a Nikon Ti-E microscope equipped with scMOS, a Leica DM 5000 Microscope with an Andor CCD camera; 40× dry objective was used for each type of microscope. Images were analyzed using Fiji software.

### 4.4. Fluorescent In Situ Hybridization

Mammalian cells were seeded onto glass coverslips and treated with NaN_3_ or cultivated under control conditions. Cells were washed with 1x PBS, fixed for 15 min with 4% PFA and cell membranes were permeabilized for 10 min using 0.5% Triton-X in PBS. The coverslips were washed in 2× SSC and incubated in a 1:1000 dilution of Alexa555-coupled oligo(dT)50 probe (100 pmol/µL, Invitrogen) in hybridization buffer (1 mg/mL yeast RNA, 20% formamide, 2 mg/mL BSA, 0.1 g/mL dextrane sulphate, 1× SSC) for 1 h at room temperature. Coverslips were sequentially washed with 2× SCC, 0.2× SCC and 1× PBS. After that, the immunostaining against G3BP was performed as stated above. The DNA was visualized using Hoechst dye at a dilution of 1:10,000. Coverslips were mounted onto microscope slides using FluoroG mounting medium (Thermo Fisher).

### 4.5. Puromycin Incorporation Assay

Prior to lysis, cells were treated with 10 µg/mL puromycin (Gibco, Life Technologies, Waltham, MA, USA) for 5 min at 37 °C, washed three times with PBS and collected in protein lysis buffer (50 mM Tris-HCl [pH 7.4], 150 mM NaCl, 15 mM MgCl_2_, 1% Triton X-100; freshly supplemented with EDTA-free protease inhibitor cocktail (Roche), 1 mM Na_3_VO_4_ and 50 mM NaF). Total protein concentrations were determined by Bradford assay. Equal amounts of cell lysates were separated by SDS-PAGE and analyzed with WB using anti-puromycin antibody (Millipore MABE343) for the visualization of puromycinylated polypeptides.

### 4.6. Western Blot Analysis

Cells were washed with PBS and collected using a cell scraper in a protein lysis buffer (50 mM Tris-HCl [pH 7.4], 150 mM NaCl, 15 mM MgCl_2_, 1% Triton X-100; freshly supplemented with EDTA-free protease inhibitor cocktail (Roche), 1 mM Na_3_VO_4_ and 50 mM NaF). After incubation for 25 min on ice, extracts were centrifuged (10,000g) in order to separate the nuclear and cytoplasmic proteins. Cytoplasmic extracts were resolved on Tris-glycine polyacrylamide (5–20% gradient or 12%) gels and transferred onto nitrocellulose membranes with a 0.2 μm pore size (Peqlab, Erlangen, Germany). Membranes were blocked in 3% BSA in PBS-A. Primary antibodies were diluted in PBS-A; membranes were incubated overnight at 4 °C and washed three times in a 150 mM NaCl solution containing 50 mM Tris [pH 7.5] and 1% Tween-20. After washing, membranes were incubated in Horseradish peroxidase–coupled secondary antibodies (Jackson ImmunoResearch Laboratories, West Grove, PA, USA) diluted in PBS. Five additional washes were performed before using Western Lightning enhanced chemiluminescence substrate (PerkinElmer, Waltham, MA, USA) for detection.

### 4.7. MitoTracker^TM^ Staining

HeLa cells were seeded on coverslips, then treated with NaN_3_ for 1h. In the last 45 min of treatment, MitoTracker^TM^ Orange CM-H_2_TMRos (Molecular Probes) was added to the culture media at a final concentration of 0.5 μM (the concentration level was chosen according to the manufacturer’s manual). Finally, the cells were fixed and prepared for microscopy as already described (4.3. Immunofluorescence analysis). 

### 4.8. Antibodies

The following primary antibodies were used in this study: mouse anti-G3BP1 (Santa Cruz, sc-81940, Santa Cruz, CA, USA), goat anti-eIF4AI (Santa Cruz, sc-14211), mouse anti-eIF4E (P-2, Santa Cruz, sc-9976), rabbit anti-eIF4G (Cell Signaling #2498), goat anti-eIF3B (Santa Cruz, sc-16377), rabbit anti-Caprin1 (Proteintech, 15112-1-AP, Chicago, IL, USA), rabbit anti-phospho(S51)-eIF2α (Cell Signaling #9721), rabbit anti-eIF2α (Cell Signaling #9722, Danvers, MA, USA), rabbit anti-4E-BP1 (Cell Signaling, #53H11), rabbit anti-phospho-4E-BP1(Thr37/46) (Cell Signaling, #236B4), rabbit anti-AMPKalpha (Cell Signaling, #2603), rabbit anti-phospho-AMPKalpha (Cell Signaling, #2535), rat anti-tubulin (Abcam, #ab6160, Cambridge, UK), rabbit anti-Trx1 (FL-105, Santa Cruz sc-20146) (WB), rabbit anti-Trx1 (Cell Signaling #2429) (IF) and mouse anti-puromycin (Millipore MABE343).

### 4.9. Statistical Analysis

Statistical data analysis and graph generation was done using R. Within an R environment, besides base-r, the ggpubr package was used to generate graphs. The statistical significance was calculated by performing Student’s t-test. All experiments have been performed at least three times.

## Supplementary Materials

The following supporting information can be downloaded at: https://www.mdpi.com/article/10.3390/ijms23105600/s1.

## References

[1] Sarkar A., Chattopadhyay S., Kaul R., Pal J.K. (2002). Lead Exposure and Heat Shock Inhibit Cell Proliferation in Human HeLa and K562 Cells by Inducing Expression and Activity of the Heme-Regulated EIF-2alpha Kinase. J. Biochem. Mol. Biol. Biophys..

[2] Holcik M., Sonenberg N. (2005). Translational Control in Stress and Apoptosis. Nat. Rev. Mol. Cell Biol..

[3] De Nadal E., Posas F. (2010). Multilayered Control of Gene Expression by Stress-Activated Protein Kinases. EMBO J..

[4] Costa-Mattioli M., Walter P. (2020). The Integrated Stress Response: From Mechanism to Disease. Science.

[5] Pakos-Zebrucka K., Koryga I., Mnich K., Ljujic M., Samali A., Gorman A.M. (2016). The Integrated Stress Response. EMBO Rep..

[6] Donnelly N., Gorman A.M., Gupta S., Samali A. (2013). The EIF2α Kinases: Their Structures and Functions. Cell. Mol. Life Sci..

[7] Eiermann N., Haneke K., Sun Z., Stoecklin G., Ruggieri A. (2020). Dance with the Devil: Stress Granules and Signaling in Antiviral Responses. Viruses.

[8] Stoecklin G., Kedersha N., Chan E.K.L., Fritzler M.J. (2013). Relationship of GW/P-Bodies with Stress Granules. Ten Years of Progress in GW/P Body Research.

[9] Mahboubi H., Stochaj U. (2017). Cytoplasmic Stress Granules: Dynamic Modulators of Cell Signaling and Disease. Biochim. Biophys. Acta Mol. Basis Dis..

[10] Kedersha N., Anderson P. (2002). Stress Granules: Sites of MRNA Triage That Regulate MRNA Stability and Translatability. Biochem. Soc. Trans..

[11] Kimball S.R., Horetsky R.L., Ron D., Jefferson L.S., Harding H.P. (2003). Mammalian Stress Granules Represent Sites of Accumulation of Stalled Translation Initiation Complexes. AJP Cell Physiol..

[12] Cherkasov V., Hofmann S., Druffel-Augustin S., Mogk A., Tyedmers J., Stoecklin G., Bukau B. (2013). Coordination of Translational Control and Protein Homeostasis during Severe Heat Stress. Curr. Biol..

[13] Jain S., Wheeler J.R., Walters R.W., Agrawal A., Barsic A., Parker R. (2016). ATPase-Modulated Stress Granules Contain a Diverse Proteome and Substructure. Cell.

[14] Buchan J.R., Yoon J.-H., Parker R. (2011). Stress-Specific Composition, Assembly and Kinetics of Stress Granules in Saccharomyces Cerevisiae. J. Cell Sci..

[15] Kedersha N., Ivanov P., Anderson P. (2013). Stress Granules and Cell Signaling: More than Just a Passing Phase?. Trends Biochem. Sci..

[16] Emara M.M., Fujimura K., Sciaranghella D., Ivanova V., Ivanov P., Anderson P. (2012). Hydrogen Peroxide Induces Stress Granule Formation Independent of EIF2α Phosphorylation. Biochem. Biophys. Res. Commun..

[17] Sama R.R.K., Ward C.L., Kaushansky L.J., Lemay N., Ishigaki S., Urano F., Bosco D.A. (2013). FUS/TLS Assembles into Stress Granules and Is a Prosurvival Factor during Hyperosmolar Stress. J. Cell Physiol..

[18] Kim W.J., Back S.H., Kim V., Ryu I., Jang S.K. (2005). Sequestration of TRAF2 into Stress Granules Interrupts Tumor Necrosis Factor Signaling under Stress Conditions. Mol. Cell. Biol..

[19] Reineke L.C., Cheema S.A., Dubrulle J., Neilson J.R. (2018). Chronic Starvation Induces Non-Canonical pro-Death Stress Granules. J. Cell Sci..

[20] Fessler E., Eckl E.-M., Schmitt S., Mancilla I.A., Meyer-Bender M.F., Hanf M., Philippou-Massier J., Krebs S., Zischka H., Jae L.T. (2020). A Pathway Coordinated by DELE1 Relays Mitochondrial Stress to the Cytosol. Nature.

[21] Guo X., Aviles G., Liu Y., Tian R., Unger B.A., Lin Y.-H.T., Wiita A.P., Xu K., Correia M.A., Kampmann M. (2020). Mitochondrial Stress Is Relayed to the Cytosol by an OMA1-DELE1-HRI Pathway. Nature.

[22] Kedersha N., Chen S., Gilks N., Li W., Miller I.J., Stahl J., Anderson P. (2002). Evidence That Ternary Complex (EIF2-GTP-TRNA _i_ ^Met^)–Deficient Preinitiation Complexes Are Core Constituents of Mammalian Stress Granules. Mol. Biol. Cell.

[23] Jovanovic B., Eiermann N., Talwar D., Boulougouri M., Dick T.P., Stoecklin G. (2021). Thioredoxin 1 Is Required for Stress Granule Assembly upon Arsenite-Induced Oxidative Stress. Food Chem. Toxicol..

[24] Fu X., Gao X., Ge L., Cui X., Su C., Yang W., Sun X., Zhang W., Yao Z., Yang X. (2016). Malonate Induces the Assembly of Cytoplasmic Stress Granules. FEBS Lett..

[25] Samluk L., Urbanska M., Kisielewska K., Mohanraj K., Kim M.-J., Machnicka K., Liszewska E., Jaworski J., Chacinska A. (2019). Cytosolic Translational Responses Differ under Conditions of Severe Short-Term and Long-Term Mitochondrial Stress. Mol. Biol. Cell.

[26] Igarashi J., Sato A., Kitagawa T., Sagami I., Shimizu T. (2003). CO Binding Study of Mouse Heme-Regulated EIF-2alpha Kinase: Kinetics and Resonance Raman Spectra. Biochim. Biophys. Acta.

[27] Chen Y., Joe Y., Park J., Song H.-C., Kim U.-H., Chung H.T. (2019). Carbon Monoxide Induces the Assembly of Stress Granule through the Integrated Stress Response. Biochem. Biophys. Res. Commun..

[28] Ishii H., Shirai T., Makino C., Nishikata T. (2014). Mitochondrial Inhibitor Sodium Azide Inhibits the Reorganization of Mitochondria-Rich Cytoplasm and the Establishment of the Anteroposterior Axis in Ascidian Embryo. Develop. Growth Differ..

[29] Bowler M.W., Montgomery M.G., Leslie A.G.W., Walker J.E. (2006). How Azide Inhibits ATP Hydrolysis by the F-ATPases. Proc. Natl. Acad. Sci. USA.

[30] Grousl T., Vojtova J., Hasek J., Vomastek T. (2021). Yeast Stress Granules at a Glance. Yeast.

[31] Khong A., Matheny T., Jain S., Mitchell S.F., Wheeler J.R., Parker R. (2017). The Stress Granule Transcriptome Reveals Principles of MRNA Accumulation in Stress Granules. Mol. Cell.

[32] Wang S., Kwon S.-H., Su Y., Dong Z. (2019). Stress Granules Are Formed in Renal Proximal Tubular Cells during Metabolic Stress and Ischemic Injury for Cell Survival. Am. J. Physiol. Ren. Physiol..

[33] Matsuki H., Takahashi M., Higuchi M., Makokha G.N., Oie M., Fujii M. (2013). Both G3BP1 and G3BP2 Contribute to Stress Granule Formation. Genes Cells.

[34] Aulas A., Caron G., Gkogkas C.G., Mohamed N.-V., Destroismaisons L., Sonenberg N., Leclerc N., Parker J.A., Velde C.V. (2015). G3BP1 Promotes Stress-Induced RNA Granule Interactions to Preserve Polyadenylated MRNA. J. Cell Biol..

[35] Advani V.M., Ivanov P. (2020). Stress Granule Subtypes: An Emerging Link to Neurodegeneration. Cell. Mol. Life Sci..

[36] Wheeler J.R., Matheny T., Jain S., Abrisch R., Parker R. (2016). Distinct Stages in Stress Granule Assembly and Disassembly. eLife.

[37] Kedersha N., Panas M.D., Achorn C.A., Lyons S., Tisdale S., Hickman T., Thomas M., Lieberman J., McInerney G.M., Ivanov P. (2016). G3BP–Caprin1–USP10 Complexes Mediate Stress Granule Condensation and Associate with 40S Subunits. J. Cell Biol..

[38] Sonenberg N., Hinnebusch A.G. (2009). Regulation of Translation Initiation in Eukaryotes: Mechanisms and Biological Targets. Cell.

[39] Taniuchi S., Miyake M., Tsugawa K., Oyadomari M., Oyadomari S. (2016). Integrated Stress Response of Vertebrates Is Regulated by Four EIF2α Kinases. Sci. Rep..

[40] Scheuner D., Song B., McEwen E., Liu C., Laybutt R., Gillespie P., Saunders T., Bonner-Weir S., Kaufman R.J. (2001). Translational Control Is Required for the Unfolded Protein Response and In Vivo Glucose Homeostasis. Molecular Cell.

[41] Mokas S., Mills J.R., Garreau C., Fournier M.-J., Robert F., Arya P., Kaufman R.J., Pelletier J., Mazroui R. (2009). Uncoupling Stress Granule Assembly and Translation Initiation Inhibition. Mol. Biol. Cell.

[42] Aulas A., Fay M.M., Lyons S.M., Achorn C.A., Kedersha N., Anderson P., Ivanov P. (2017). Stress-Specific Differences in Assembly and Composition of Stress Granules and Related Foci. J. Cell Sci..

[43] Nadezhdina E.S., Lomakin A.J., Shpilman A.A., Chudinova E.M., Ivanov P.A. (2010). Microtubules Govern Stress Granule Mobility and Dynamics. Biochim. Biophys. Acta Mol. Cell Res..

[44] Horn A., Van der Meulen J.H., Defour A., Hogarth M., Sreetama S.C., Reed A., Scheffer L., Chandel N.S., Jaiswal J.K. (2017). Mitochondrial Redox Signaling Enables Repair of Injured Skeletal Muscle Cells. Sci. Signal..

[45] Harvey J., Hardy S.C., Ashford M.L.J. (1999). Dual Actions of the Metabolic Inhibitor, Sodium Azide on Katp Channel Currents in the Rat CRI-G1 Insulinoma Cell Line: Azide Activates K _ATP_ Channels. Br. J. Pharmacol..

[46] Muneyuki E., Makino M., Kamata H., Kagawa Y., Yoshida M., Hirata H. (1993). Inhibitory Effect of NaN3 on the F0F1 ATPase of Submitochondrial Particles as Related to Nucleotide Binding. Biochim. Biophys. Acta.

[47] Hofmann S., Cherkasova V., Bankhead P., Bukau B., Stoecklin G. (2012). Translation Suppression Promotes Stress Granule Formation and Cell Survival in Response to Cold Shock. Mol. Biol. Cell.

[48] Huang Q., Zhou H.J., Zhang H., Huang Y., Hinojosa-Kirschenbaum F., Fan P., Yao L., Belardinelli L., Tellides G., Giordano F.J. (2015). Thioredoxin-2 Inhibits Mitochondrial Reactive Oxygen Species Generation and Apoptosis Stress Kinase-1 Activity to Maintain Cardiac Function. Circulation.

[49] Oka S.-I., Chin A., Park J.Y., Ikeda S., Mizushima W., Ralda G., Zhai P., Tong M., Byun J., Tang F. (2020). Thioredoxin-1 Maintains Mitochondrial Function via Mechanistic Target of Rapamycin Signalling in the Heart. Cardiovasc. Res..

[50] Zaobornyj T., Mazo T., Perez V., Gomez A., Contin M., Tripodi V., D’Annunzio V., Gelpi R.J. (2019). Thioredoxin-1 Is Required for the Cardioprotecive Effect of Sildenafil against Ischaemia/Reperfusion Injury and Mitochondrial Dysfunction in Mice. Free. Radic. Res..

[51] Johannsen D.L., Ravussin E. (2009). The Role of Mitochondria in Health and Disease. Curr. Opin. Pharmacol..

