# Supplementary material for: Mitochondrial Inhibition by Sodium Azide Induces Assembly of eIF2α Phosphorylation-Independent Stress Granules in Mammalian Cells
Source: Int J Mol Sci. 2022 May 17;23(10):5600. doi: 10.3390/ijms23105600 (PMC9142010; doi:10.3390/ijms23105600)
Supplement: Supplementary file 1 [file ijms-23-05600-s001.zip › ijms-1665261-SI.pdf]

Supplementary files

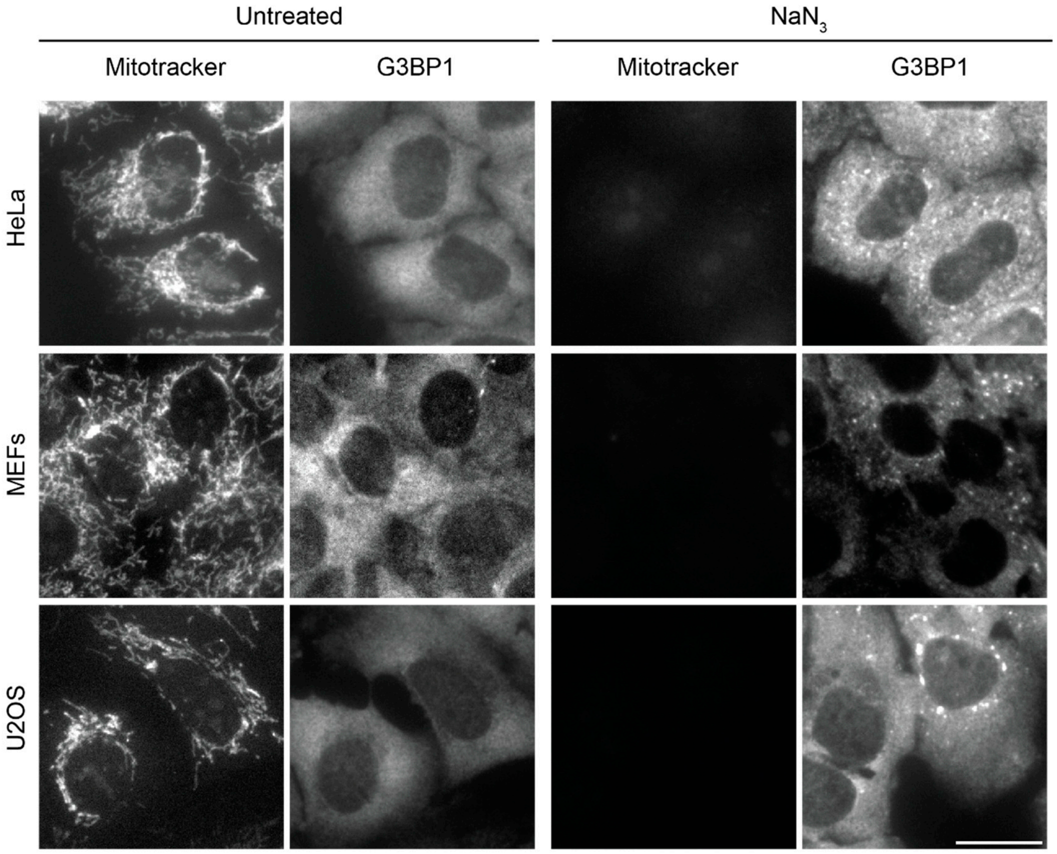

Figure S1. NaN_3 treatment leads to mitochondrial inhibition. HeLa, MEFs and U2Os cells were left untreated or treated with 0.5% v/v (76 mM) NaN_3 for 1h. 45 minutes before fixation, Mitotracker probe (CM-H_2TMRos) was added to the culture medium. After fixation, cells were stained against G3BP1 and analyzed by microscopy. Scale bar = 20 μm.

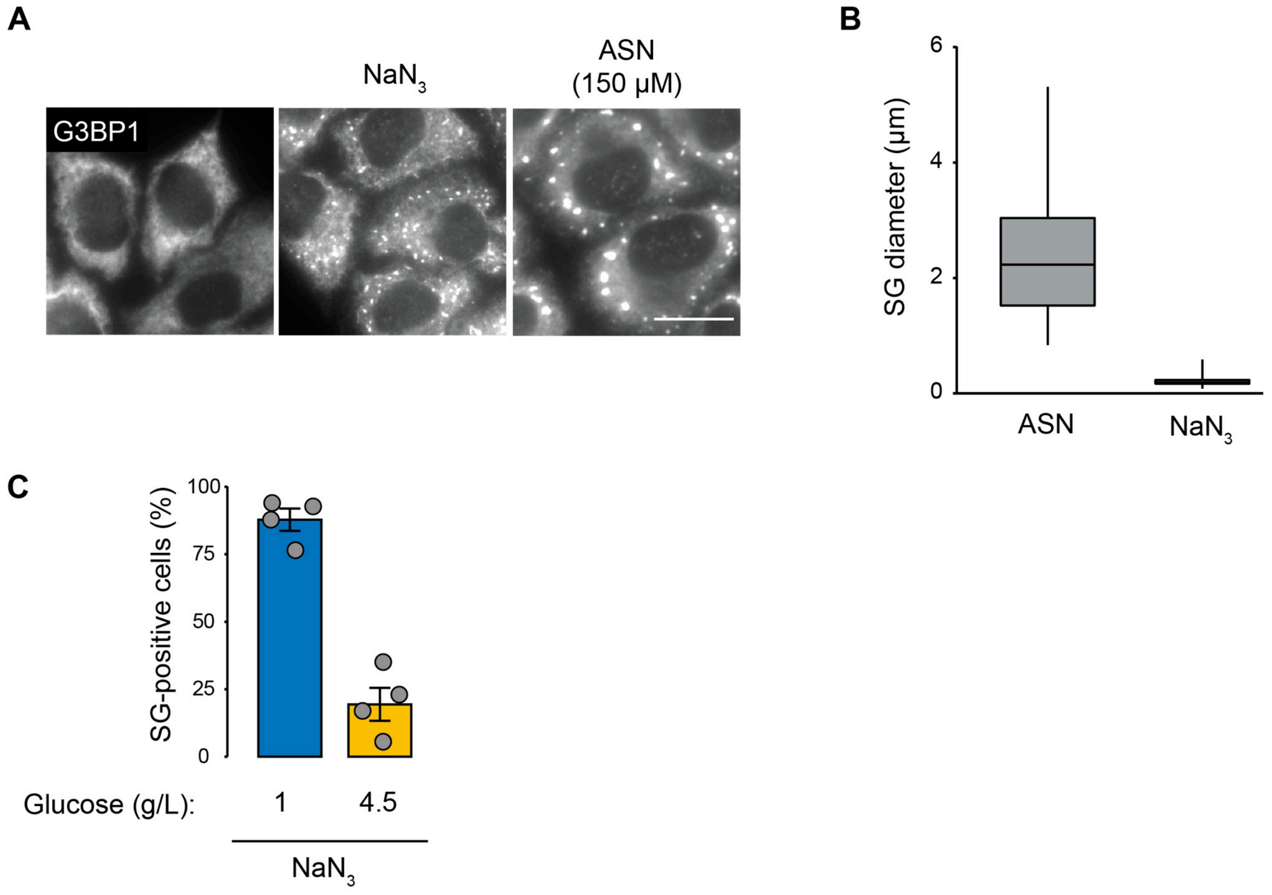

Figure S2. NaN_3-induced SGs are smaller than common SGs induced by ASN. **A,** HeLa cells were left untreated or treated with 150 µM ASN for 1h or 76 mM NaN_3 for 2h. Cells were fixed, stained against G3BP1 and analyzed by microscopy. Scale bar = 20 µm **B**, Diameters of SGs in HeLa cells were measured using Fiji software. Results are presented as a boxplot. Line in the box refers to the median value. Data analysis is based on 202 measurements of SG diameter for each group. **C**, Quantification of SGs formed in HeLa cells treated with NaN_3 in high (4.5 g/L) and low (1 g/L) glucose medium. The graph shows mean value ± SD (n = 4 independent experiments, approximately 100 cells were analyzed per experiment and condition).

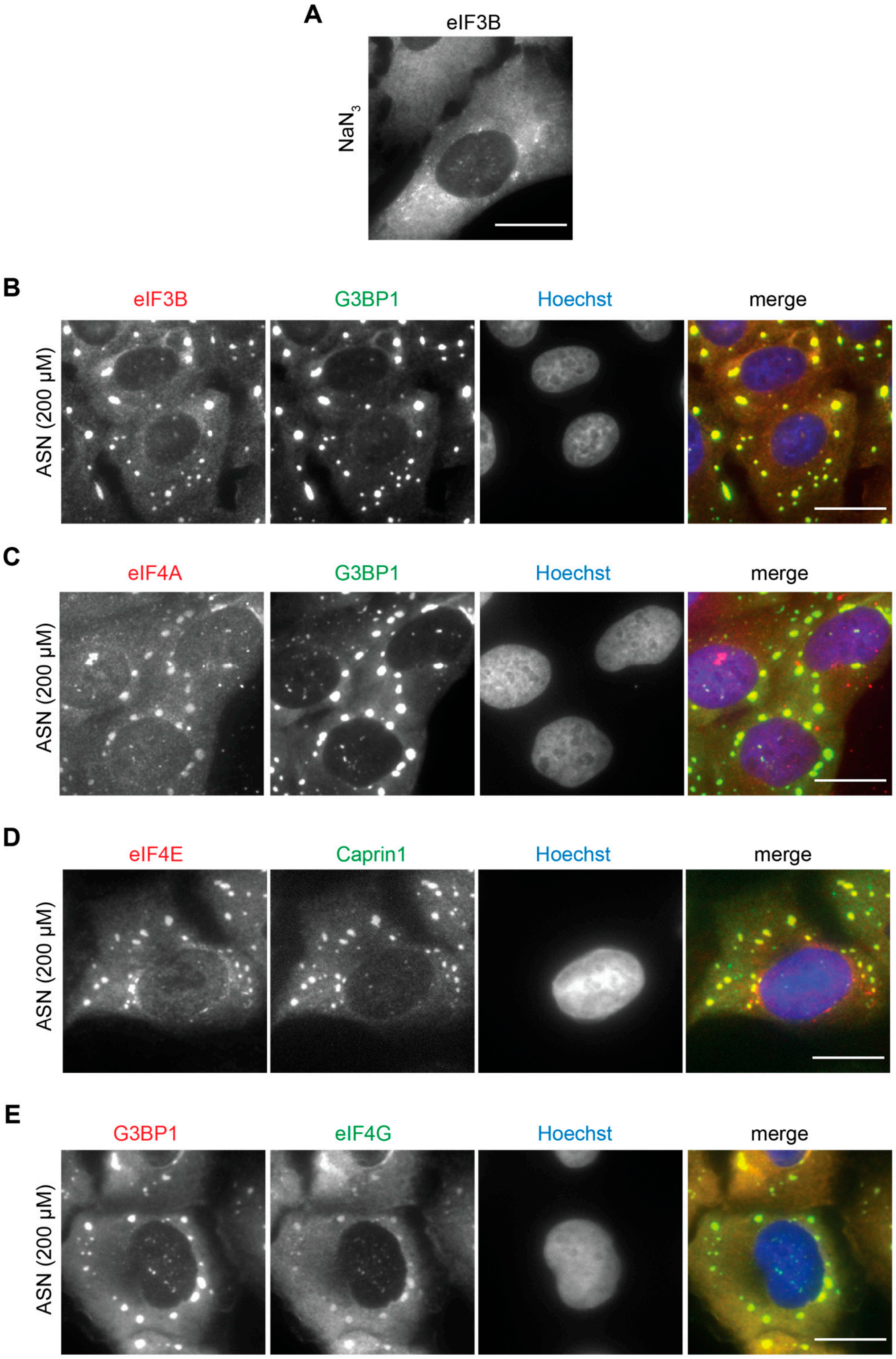

Figure S3. Characterization of NaN_3- and ASN-induced SGs. **A,** Single immunostaining of eIF3B in NaN_3-treated U2OS cells. Scale bar = 20 µm. **B,** U2OS were treated with ASN and immunostained against G3BP1 together with eIF3B. **C,** Co-immunostaining of eIF4A and G3BP1 in U2OS cells treated with NaN_3 (76 mM). Scale bar = 20 µm. **D**, Co-immunostaining of Caprin1 and eIF4E in U2OS cells treated with ASN. Scale bar = 20 µm. **E**, Co-immunostaining of eIF4G and G3BP1 in U2OS cells treated with NaN_3. Scale bar = 20 µm.

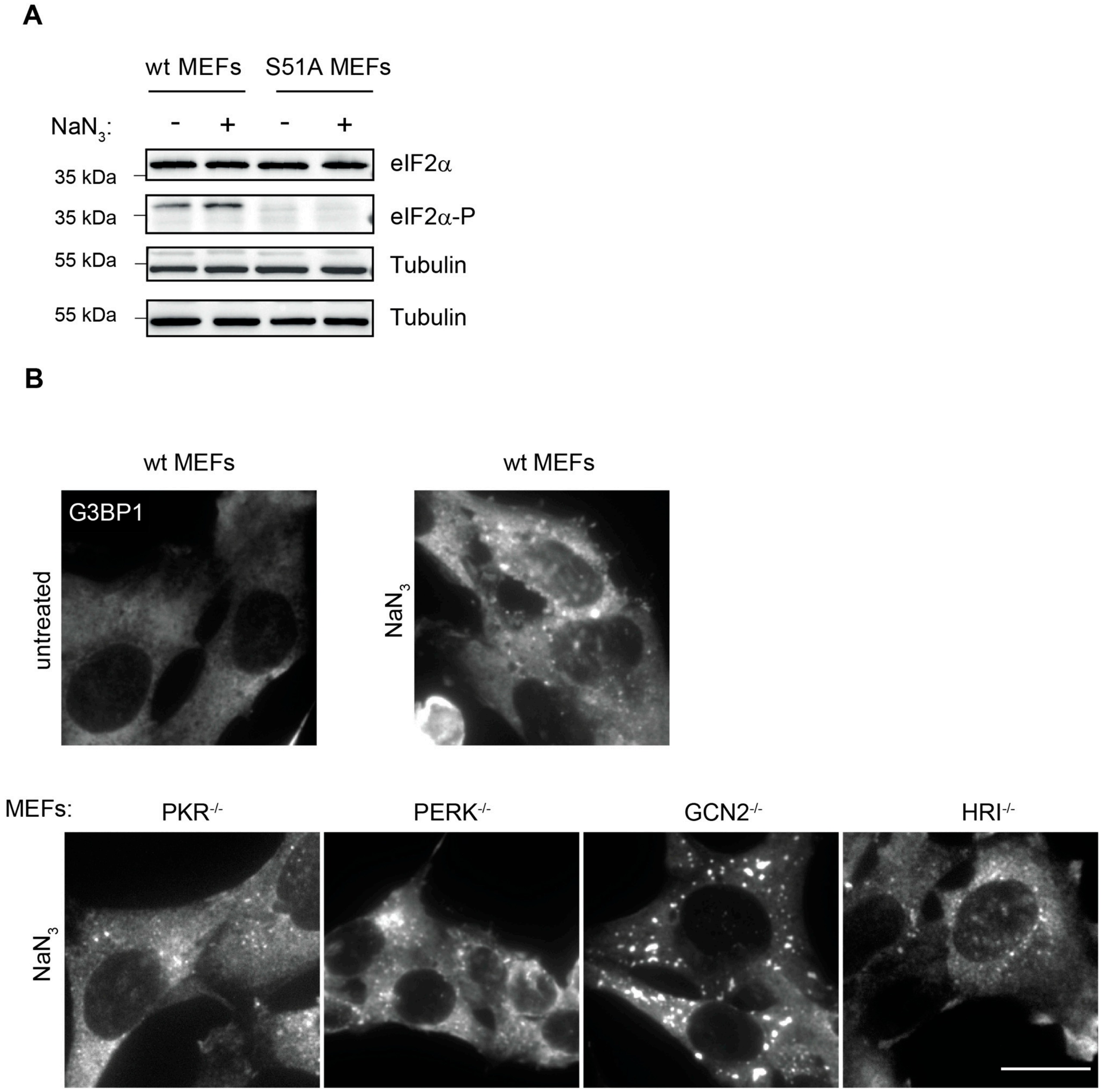

Figure S4. eIF2a kinases do not mediate SG formation upon exposure to NaN_3. **A,** S51A and wt MEFs were treated with NaN_3 (76 mM) for 1h or left untreated, lysed and analyzed for the presence of eIF2α phosphorylation using WB. **B,** Different MEF mutants, deficient for the individual stress kinases, were treated with NaN_3 (76 mM), fixed and prepared for IF. Subcellular localization of G3BP1 was used for detection of SGs. Scale bar = 20 μm.
